# Study of Polyurethane Microplastics Removal from Water Using Smart Installation

**DOI:** 10.3390/polym18121513

**Published:** 2026-06-17

**Authors:** Daniela Simina Stefan, Gheorghe Pauna, Andreea Alexandra Barbu, Rachid Aziam, Ana Iulia Stefan

**Affiliations:** 1 Department of Analytical Chemistry and Environmental Engineering, Faculty of Chemical Engineering and Biotechnology, National University of Science and Technology Politehnica of Bucharest, 1-7 Polizu Street, 011061 Bucharest, Romania; gelupauna@yahoo.com; 2 Faculty of Chemical Engineering and Biotechnology, National University of Science and Technology Politehnica of Bucharest, 011061 Bucharest, Romania; andreea.barbu0108@stud.chimie.upb.ro; 3 Higher School of Education and Training (ESEF), Ibnou Zohr University, Agadir 80000, Morocco; rahid.aziam@edu.uiz.ac.ma; 4 Regina Maria-Dental Hospital, Cozia 71 Street, 300425 Timisoara, Romania

**Keywords:** polyurethane microplastics, coagulation, flocculation, sedimentation, smart installation, efficiency

## Abstract

Microplastics, MPs, plastic particles with dimensions between 0.1 and 5 mm, represent an important environmental pollutant. The removal of microplastics from natural and wastewater is a challenging research topic. In this regard, high-performance technical solutions must be identified, which can be based on existing treatment and purification technologies, to ensure their removal at concentration values in accordance with the legislation in force. In this study, the efficiency of removing some fractions of polyurethane microplastics, with dimensions smaller than 500 µm, from aqueous synthetic solutions with a concentration of 0.2 g L^−1^, i.e., around 175 NTU, was evaluated. In the first stage of the study, the doses of coagulants and flocculants effective for the removal of microplastics were identified through the Jar Test. The variation in turbidity and their removal efficiencies were evaluated in the presence of classic coagulants, such as aluminum sulfate, Al_2_(SO_4_)_3_·18H_2_O, SA; iron sulfate (ferrous sulfate), FeSO_4_, IS; polyaluminum chloride, [Al_2_(OH)_n_Cl_6−n_], PAC; Aloe Vera, AV, a flocculant; and activated carbon, AC, of the Norit GAC 830 W type. Classic coagulants, such as aluminum sulfate, have a good efficiency in removing microplastics, being able to provide a residual turbidity in the range of 6–10 NTU after a retention time of 50–60 min. In the second stage of the study, the removal efficiency of microplastics was tested using a laboratory pilot plant—called in the study the Smart Decantation-Filtration System, SDFS. The efficiency of the decanter was studied using Response Surface Methodology (RSM) to identify mathematical models that characterize the influence of key process variables: flow rate (A), microplastic size (B) and aluminum sulfate concentration (C) on microplastic removal efficiency. Sedimentation in the specially constructed decanter can raise the optimal value of the removal efficiency of polyurethane microplastics to 98.98%, and filtration can ensure an efficiency that reaches over 99.5%. Through this research, we aimed to identify viable solutions that can be applied to remove microplastics, MPs, from natural and wastewater. A novel element is the fact that we chose to study the removal of polyurethane, which is studied little in the literature. We identified the optimal doses of coagulants and flocculants that help sedimentation of MPs. The efficiency of an installation called Smart Decantation-Filtration System, specially designed to ensure increased efficiency in the removal of microplastics, was determined. The results obtained were encouraging.

## 1. Introduction

Globally, the demand and manufacturing of plastics have shown significant growth. Plastics products have become an integral part of today’s civilization, due to their convenience, accessibility, functional properties and low cost. Their use is driven by several variables such as population growth, increasing urbanization, and advances in technology [1].

The production of plastics started around 1950 at two million tons, reaching 436.66 million tons traded in 2022, and is estimated to reach 884 Mt in 2050 with an accumulation of 4725 Mt between 2000 and 2050. Each year, an estimated 4 to 12 million tons of plastics enter the oceans, and by 2050 there may be more plastics than fish in the sea [2,3,4]. The accelerated growth of plastic production has generated significant problems regarding waste management and their impact on the environment ecosystems and human health [1].

Plastics can be described as “synthetic” (non-renewable) or “bio” (renewable) plastics. Synthetic plastics are extracted from crude oil, natural gas or coal. Biological plastics are made from renewable product materials such as starch, carbohydrates, fats and vegetable oils [5].

The most common plastics include polypropylene (PP), polyethylene (PE), low-density polyethylene (LDPE), high-density polyethylene (HDPE), polyethylene terephthalate (PET), polystyrene (PS), polyurethane (PU) and polyvinyl chloride (PVC) [6]. Polyethylene is one of the most persistent plastics from an ecological point of view and its accumulation has created a serious pollution concern [7].

Around 76% of the total plastics production is collected as waste, of which 12% is incinerated, 79% is landfilled or released into the environment and only 9% is recycled [2].

It is estimated that between 5.3 and 14 million tons of plastic waste are dumped along the coasts every year, 10% of which ends up in the hydrosphere and accumulates over time [6]. Plastics are exposed to a gradual decomposition process that changes the chemical and physical structures of polymers, which results in the formation of small plastic fragments [8,9]. These molecules can have the following sizes: megaplastic (>50 cm), macroplastic (5–50 cm), mesoplastic (0.5–5 cm) and microplastic (0.1–5 mm) [10,11].

The term “microplastics”, MPs, was first used in 2004, in a study that mentioned their presence in the marine environment, both in beach sediments and inside marine organisms [12]. They are characterized as being non-biodegradable and long durable in the environment [13,14].

Microplastic particles are classified into two groups: primary and secondary [15]. Primary microplastics are manufactured for industrial purposes or personal applications and include plastic granules used in the cosmetics industry, and personal care products such as facial cleansers and toothpaste. Secondary microplastics result from the degradation of plastics in environment, by mechanical, chemical or biological actions [15,16]. By their shape, microplastics can be classified as: microbeads, granules, fibers, films, fragments and filaments [17]. Color is an important factor affecting aquatic organisms. Microplastics present in seawater samples and sediments are colorless or white; multicolored or black microplastics are less common. Aquatic animals may confuse their natural food with microplastics because of the similarity of colors [18].

Microplastics are present in a variety of environments around the world, including air, natural water (lakes, rivers, seas and oceans), food, drinking water, wastewater, agriculture land and soil. Microplastics have been found in bottled water and groundwater sources [19,20].

The life cycle of microplastics starts with the release of primary or secondary microplastics and plastic waste (in continuous degradation) into wastewater, or into atmospheric, aquatic and terrestrial ecosystems; it continues with their transportation under the action of the atmospheric currents, rainfall, surface runoff and ocean circulation and they disperse around the Earth, concentrating in the final in aquatic systems. The concentration of microplastics, as well as their distribution in sediments and water, are influenced by location, wind intensity and water flow [21,22].

After they accumulate in various parts of the environment, microplastics have two main destinations: they either fragment and sediment or are ingested by aquatic organisms and enter the food chain. The destination of microplastics is the body of a human, the final consumer [23,24].

The significant presence of microplastics in aquatic environments has generated intense concern about their harmful effects on marine life. These particles are detected in the entire water column, from surface waters to the deep sea and also in the atmosphere and soil, thus causing a substantial risk to the aquatic life [15]. Around 70% of marine plastic waste is deposited in sediments, 15% is found in coastal areas and the rest float on the surface of seawater [25].

Microplastics act as carriers of dangerous chemicals including heavy metals, organic pollutants and plasticizers. Ingestion of microplastics by marine organisms disturbs their physiological functions and can cause mechanical damage in the digestive tract, which results in inflammation [26]. Microplastics affect soil characteristics; they can become attached to dust or soil particles, affecting the quality of the soil and having negative effects on the growth of the plants [27,28].

The environmental fate of microplastics is a continuing concern to identify how it can influence human health [29]. Ingestion, inhalation and dermal contact are the most common routes of exposure to microplastics. Ingestion of food and drinking water is considered the most important route [30]. Seafood is the most common source of food that contains high levels of microplastics [31,32]. Microplastics that enter the body can interact with histologic structures, affect cellular structure and function, and can be transported via the bloodstream to vital organs, potentially including the heart. They cause changes in the gut microbiota and induce an imbalance between healthy and harmful microorganisms, negatively impacting the gastrointestinal tract and the entire body [33].

These cause alterations in the intestinal microbiota and induce a disequilibrium between healthy and harmful microorganisms, having a negative impact on the gastrointestinal tract and the entire body [33]. The respiratory system is affected by the production of oxidative stress in the respiratory airways and the lungs, which causes cough, sneezing and difficulty breathing due to inflammation, as well as dizziness due to low oxygen concentrations in the blood [33].

Microplastics also accelerate hemolysis and the formation of a molecule that stimulates inflammation. These negative consequences vary depending on the level of exposure to these particles, and the susceptibility of the human body [34]. As can be seen, the negative effects of microplastics on the environment and humans are multiple and all implications are not yet known. Additives used to improve the quality of plastics, such as bisphenol A, are released and absorbed into the body, and are responsible for endocrine and reproductive system disruption [33].

Their recovery from the environment is very difficult due to their small size, low specific gravity and compositional variety. The microplastics removal technologies from natural water and wastewater use physical, chemical and biological methods. The physical methods consist of filtration by membranes (microfiltration, ultrafiltration, and reverse osmosis) [35,36], filtration by different media like quartos sand, zeolites, activated carbon and biochar, etc. [37,38,39], or sedimentation associated with coagulation–flocculation [40], magnetic separations [41], etc.

Chemical methods can include oxidation processes, for example advanced oxidation processes like Photo-Fenton, photo-catalysis and UV/H_2_O_2_ [42,43,44]. Other chemical processes are very well represented by coagulation–flocculation followed by sedimentation [45,46,47], the removal efficiency of MPs varies between 40.5 and 54.5% and even 77.83% in water treatment plants, DWTP [48]. Biological methods consist of biodegradation using mainly bacteria and fungi [49,50].

The main coagulants that have been used are iron salts (ferrous sulfate, ferric chloride, etc.) and aluminum (aluminum sulfate, aluminum chloride, polyaluminum chloride, etc.), as well as flocculants such as polyacrylamide lysozyme monomers, Aloe Vera, etc. [51,52]. For optimum doses, the reduction efficiency can reach 72% for turbidity, and 91% for suspended matter. Aloe Vera, a new flocculant, contains tannins, saponins, flavonoids, anthracene derivatives, quinone derivatives and proteins. Therefore, Aloe Vera gel contains chemical compounds that can agglomerate fine particles in suspension, promoting the formation of large, sedimentable floccules. The use of Aloe Vera is a possible alternative to chemical flocculants for the treatment of water with microplastics [52].

The study of the removal efficiency of microplastics by coagulation–flocculation using common coagulants is justified, because these methods can be applied to existing wastewater treatment technologies, reducing their removal costs. The behavior of microplastics in water is similar to those of colloidal compounds. They form dielectric layers, the diffuse electric double layer, described by the model Derjaguin–Landau–Verwey–Overbeek (DLVO). The electrical charge on the surface depends on the salt content, pH, and the chemical composition of the microplastic (additives, pigments, etc.). Usually, under normal conditions, the microplastics in water are negatively charged [53].

This study focused on removing polyurethane from water because it is one of the most used plastic materials and at the same time one of the most dangerous, being the fifth out of 55 polymers evaluated according to the Hazard Classification of Monomers [54]. Polyurethane is obtained by a chemical reaction between polyols (soft part) and diisocyanates (hard part), two lichids [55].

Polyurethanes are materials with special properties, variable density, open cell structure, good air permeability, good dimensional stability, flexibility, elasticity, good adhesion and others, and at the same time have the ability to form composites with nanomaterials, such as carbon nanotubes, graphene derivatives, clay, and silica, for improving qualities, modifying properties, etc. [54].

They can be used in various industrial and agricultural applications because they can be found in the form of low-density flexible foam used in construction, the car manufacturing industry for upholstery and car seats, landscaping with synthetic vegetal layers, and construction for roofs and walls. They can also be in the form of low-density elastomers used in the footwear industry for smart soles and other components. They can also be used as flexible plastic materials for tapes, belts, protective suits, clothing and accessories, packaging, etc. Composite materials with polyurethanes can form high-density materials: hard polyurethanes that are used as components of electronic instruments, for the car manufacturing industry, or molded parts with various applications in all fields (furniture, agriculture, electronics and household appliances, etc.) [56].

The present study focused on the removal of polyurethane microplastics with sizes smaller than 500 µm from aqueous synthetic solutions at a concentration of 0.2 g L^−1^, around 175 NTU. In the first stage of the study, tests were performed to identify the optimal doses of coagulants and flocculants for microplastic removal, using the classical method, the Jar Test. At this stage, attention was directed towards analyzing the variation in turbidity and their removal efficiency in the presence of classical coagulants such as: aluminum sulfate, Al_2_(SO_4_)_3_·18H_2_O, SA; iron sulfate (ferrous sulfate), FeSO_4_·7H_2_O, IS; polyaluminum chloride, [Al_2_(OH)_n_Cl_6−n_], PAC; Aloe Vera gel, AV, flocculant; and activated carbon, AC, of the Norit GAC 830 W type. In the second phase of the study, the efficiency of the Smart Decantation-Filtration System, SDFS, specifically designed for the removal of microplastics, was determined. The efficiency for the removal of microplastics of the above-mentioned sizes from synthetic solutions was tested with simple microplastics only, but also with the reactives in the optimal doses identified according to the Jar Test. The efficiency of the decanter was studied using Response Surface Methodology (RSM) to identify mathematical models that characterize the influence of key process variables: flow rate (A), microplastic size (B) and aluminum sulfate concentration (C) on microplastic removal efficiency.

## 2. Materials and Methods

### 2.1. Preparation of Materials

#### 2.1.1. Microplastics Characterization and Work Condition

The microplastic samples used in the study were obtained from polyurethane foam. The polyurethane foam used in the study is a single-component expandable foam for assembly, insulation and gap filling, a foam very often used in construction, obtained from petroleum compounds, manufacturer Bostik Den Braven, Buftea, Romania. After spraying the contents of the spray, a foam was formed that was left to harden for 24 h. After hardening, the foam was frozen at −10 °C for 24 h. Fine particles of various sizes of polyurethane were produced by grinding using a ceramic grind with pestle. For the separation into granulometric fractions, an automatic sieving system Laboratory Test Sieve, Retsch, Haan, Germany with a mesh size of 500 µm, 300 µm, 200 µm, 100 and 50 µm was used.

In the study, for determining the optimal dose through the Jar Test, the particle size fractions that passed the 500 µm sieve and the one that remained on the 200 µm sieve, named D2, and the fraction that passed the 100 µm sieve, named D1, were used. For the optimization of the process in the Smart Decantation-Filtration System, SDFS, by Response Surface Methodology (RSM), three fractions were used: the fraction that passed the 100 sieve and was estimated as an average size of 50 µm; the fraction that passed the 300 µm sieve and remained on the 100 µm and was considered to have an average diameter of 200 µm; the fraction that passed the 500 µm sieve and remained on the 200 µm and was considered to have an average size of 350 µm. Working in this way, we covered the entire range of microparticle sizes.

#### 2.1.2. Chemical Reagents

The coagulants used in this study, aluminum sulfate, ferrous sulfate and aluminum polychloride, compounds commonly used as coagulants in water treatment processes, were delivered by the company Sigma Aldrich, Burlington, MA, USA. The coagulant doses used were in the range 0–60 mg L^−1^.

Aloe Vera gel, AV, was used to increase the efficiency of the sedimentation process. In the literature studies, it has been shown that, even in low dosages, AV can remove highly loaded water from suspended solids, and thus turbidity. The Aloe Vera gel used in the study was taken from an Aloe Vera plant in the research laboratory. Aloe Vera was cut, the gel was removed, ground, filtered and dosed into synthetic solutions containing microplastics. The doses of Aloe Vera used were 1–3 g L^−1^.

Norit GAC 830 W-type activated carbon, AC, delivered by Norit Americas, Marshall, TX, USA was used as an adsorbent, which is recognized for its high efficiency in physical adsorption processes, due to its large specific surface area and high porosity [57,58]. The dose of activated carbon was 0.5 g L^−1^. The technical specifications of Norit GAC 830 W activated carbon are summarized in Table 1.

#### 2.1.3. Smart Decantation-Filtration System, SDFS

**Figure 1:**
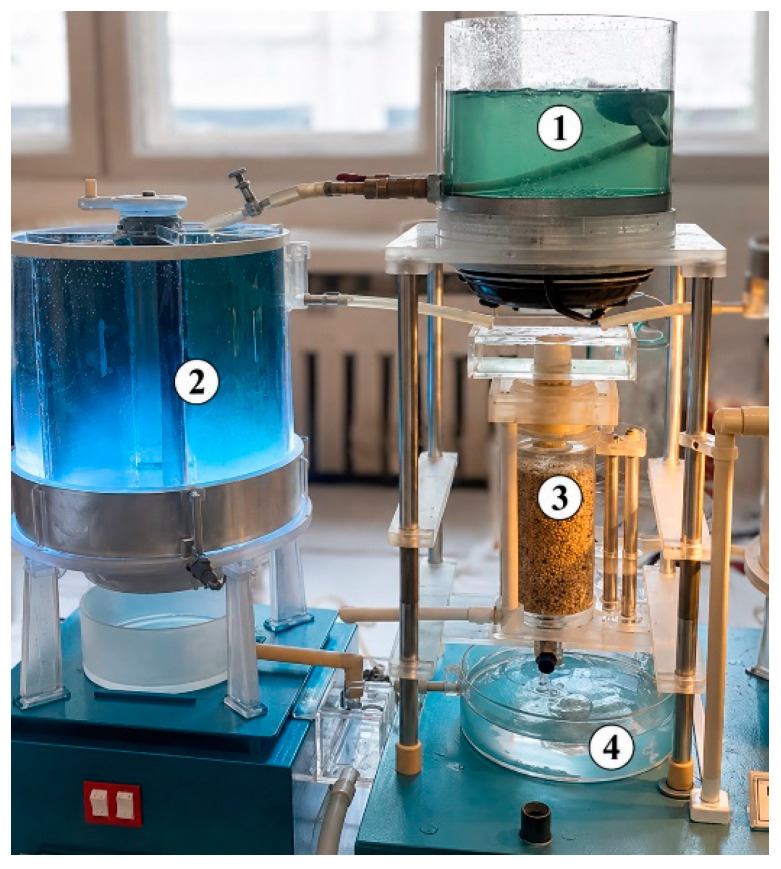
Smart Decantation-Filtration System, SDFS.

To study the removal efficiency of polyurethane microplastics, a Smart Decantation-Filtration System, SDFS, made up by decantation and filtration units, was used, as shown in Figure 1. This installation was specifically designed for the removal of microplastics. It operates continuously and consists of 4 components: a raw water tank, 1, a multicompartment decanter, 2, a quartz sand filter, 3, and a treated water tank, 4. The decanter has an intelligent design consisting of several compartments arranged in series to ensure a longer sedimentation time, at least equal to the optimum obtained in the Jar Tests.

Synthetic solutions with microplastics, as well as microplastics with SA, were introduced into the raw water tank and dosed at variable flow rates, between 1.0 and 4.0 L h^−1^ in the decanter. The synthetic solutions were passed through the decanter compartments (2).

Initial turbidity was measured in the suspension in the raw water tank (1), and turbidity at the output from the decanter was measured from the supernatant (10 mm above the water surface), the depth from which the filter feed line starts. The water from the supernatant was finally introduced into the quartz sand filter (3). The treated water output of the filter was stored in the treated water tank (4). The final turbidity was determined in the water sample from the treated water tank.

#### 2.1.4. FTIR Spectrum of Microplastics

**Figure 2:**
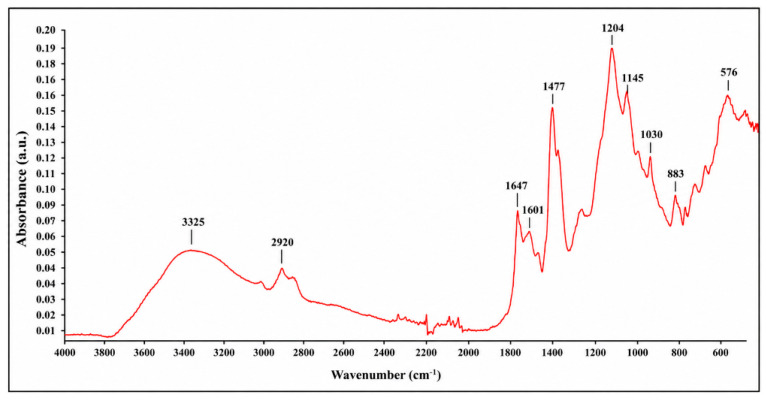
FTIR spectrum of microplastics used in the research.

Fourier transform infrared spectroscopy (FT-IR) using a Nicolet IS50FT-IR spectrometer equipped with a DTGS detector, produced by Nicolet, Wilmington, MA, USA, that provides information with high sensitivity in the range of 4000 cm^−1^ and 100 cm^−1^, at a resolution of 4 cm^−1^, were used. The IR spectrum is shown in Figure 2.

#### 2.1.5. The Jar Test

The Jar VELP Scientifica F105A0117 FP4 test, priodused by VELP Scientific, Inc., New York, NY, USA was used to determine the optimal doses of reagents required for the removal of polyurethane microplastics from synthetic solutions. The Jar Test contains four stirring rods and four glasses of one liter. The stirring rate and time can be adjusted using the buttons located on the front panel.

Turbidity was determined using a HACH 2100P 150 turbidimeter., produced by Hach Company, Loveland, CO, USA. Synthetic solutions were prepared with a concentration of 0.2 g L^−1^. The numerical values of the initial turbidity ranged from 165 to 180 NTU, the working temperature was 20 °C and the initial pH of the water was 7.2.

**Figure 3:**
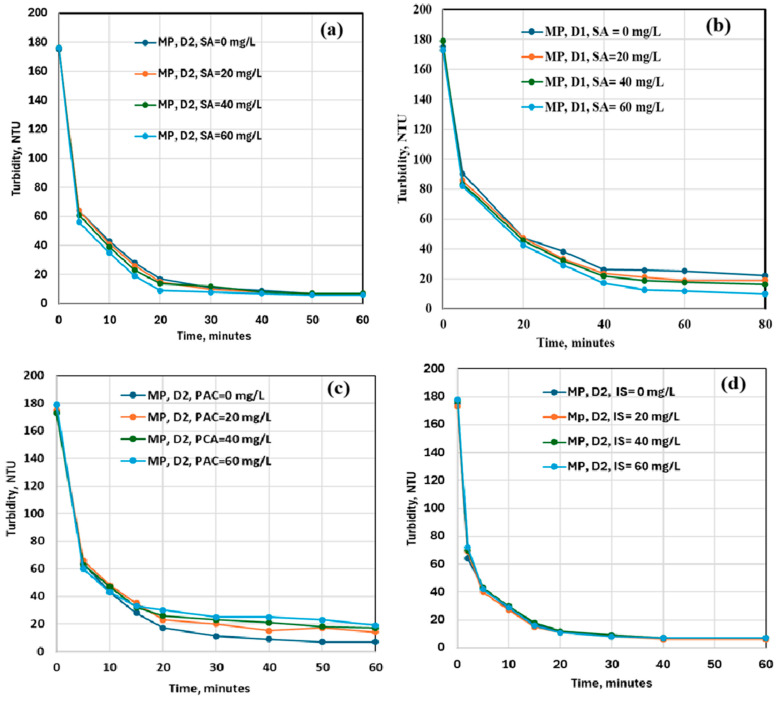
Turbidity variation in microplastics of various sizes in the presence of different coagulants: (**a**) Sulfate of aluminum, SA, D2. (**b**) Sulfate of aluminum, D1. (**c**) Polychloride of aluminum, PAC, D2. (**d**) Iron sulfate, IS, D2.

**Figure 4:**
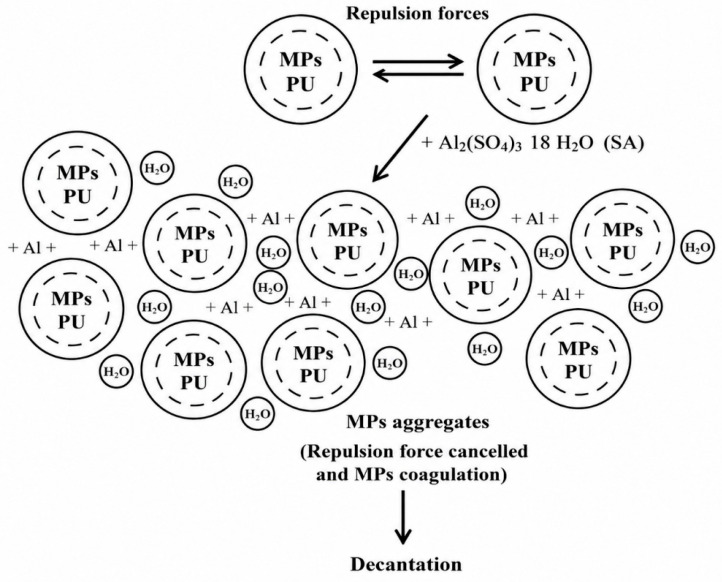
The possible mechanism of MPs from PU removal by coagulation under influence of SA.

**Figure 5:**
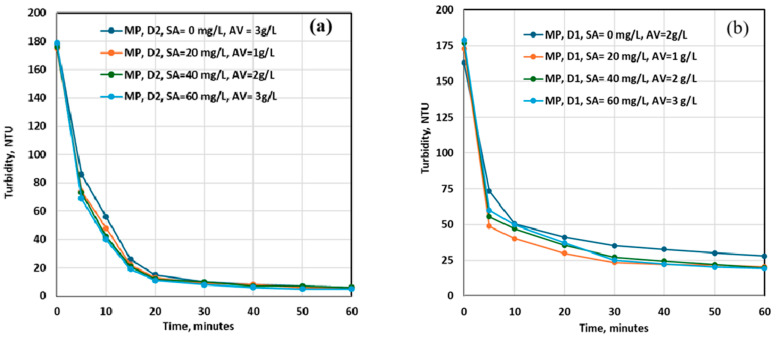
Turbidity variation in microplastics of various sizes in the presence of aluminum sulfate and Aloe Vera: (**a**) for D2 size, and (**b**) for D1 size.

The following coagulants were used: aluminum sulfate, SA; iron sulfate (ferrous sulphate), IS; aluminum polychloride, PAC—with concentration values ranging between 0 and 60 mg L^−1^, in accordance with the literature data [59]; Aloe Vera, AV, with doses between 1 and 3 g L^−1^; and activated carbon 0.5 g L^−1^, randomly chosen. The samples were stirred using the Jar Test for 1 min at 100 rpm, and 14 min at 50 rpm, in accordance with the standard procedure. After reading the initial turbidity, the samples were allowed to settle. The retention time was 60 min, during which the turbidity in the supernatant was measured at different time intervals. Graphical representations of the variation in turbidity removal efficiency with time are shown in Figures 3–5.

#### 2.1.6. Design of Experiments Based on the Box–Behnken Approach

Response Surface Methodology (RSM) is a commonly used statistical approach designed to fine-tune experimental setups and assess how various factors impact a specific outcome. This technique facilitates the construction of predictive models that incorporate both the separate and the combined influences of independent variables [60,61]. In this research, the polyurethane microplastic removal in the decanter process was optimized using the Box–Behnken Design (BBD) integrated within the Response Surface Methodology (RSM) framework, implemented through Design Expert 13 software. This approach is especially efficient in reducing the number of required experiments, while enhancing the precision of the predictive model [62,63]. A three-level factorial design (−1, 0, +1) was used to evaluate the effects of key process variables: flow rate (A), microplastic size (B), and aluminium sulphate concentration (C). The coded levels and corresponding experimental values for each factor are presented in Table 2.

Mathematical modeling aims to establish a functional relationship, denoted as

Y = f(x_1_, x_2_, x_3_, …, x_n_) (1)

where Y denotes the dependent outcome, and x_1_ to x_n_ represent the independent variables or contributing factors. This model is classified as deterministic, meaning the result is strictly determined by the inputs, without accounting for randomness or measurement uncertainty. Often, the response is estimated using a second-degree polynomial equation, offering a versatile yet organized method for representing non-linear interactions among variables.

Y (%) = β_0_+ Σ β_i_x_i_ + Σ β_ij_ x_ij_ + Σ β_ii_ x_i_^2^ + ε (2)

Y (%) represents the estimated response and serves as the key variable under investigation. It is measured with defined accuracy. The term β_0_ refers to the intercept or mean value of the response, while β_i_, β_i__j_, and β_ii_ denote the model’s coefficients, which are initially unknown and must be derived from experimental observations. The variables x_i_ and x_j_ are the independent factors influencing the response, and ε accounts for the residual error in the model [60,61].

## 3. Results and Discussions

### 3.1. FTIR Microplastics Characterization

One of the most effective methods for chemical characterization is Fourier transform infrared spectroscopy (FTIR). The IR spectrum is characterized by adsorption peaks that are specific to the vibrations of the constituent atoms of the material. The IR spectrum of the unknown material from which the microplastics are made was compared with the IR spectra of some known plastic materials [64,65]. The FTIR analysis is shown in Figure 2.

From the analysis of Figure 2, we can observe the characteristic peaks. The characteristic vibrations of the peaks were highlighted by Villegas-Camacho, O. et al. [65] as follows: 3325 cm^−1^ is specific to the vibration of the urethane group (-NH-COO-) in the heavy segment (diisocyanate); 2920 cm^−1^ is attributed to the aromatic C-N stretching; 1647 cm^−1^ is attributed to the stretching of the C=O bond of the ester group; 1601 and 1477 cm^−1^ indicate the presence of the C=C stretching vibration of the benzene rings; 1204 cm^−1^ and 1030 cm^−1^ are evidenced the C–O bond of the ester group; 883 cm^−1^ is characteristic of the C–H bending vibration of the substituted benzene ring; and 576 cm^−1^ is specific for substituted C–H bonds. All these signals confirm the polyurethane properties of the plastic.

### 3.2. The Jar Test—Determining the Optimal Doses of Reagents

#### 3.2.1. Identification of the Optimal Doses of Coagulants

Studies in the literature mention that microplastics are charged with a negative charge in water. Thus, they behave like colloidal particles of clay and humic compounds and repel each other, thus keeping them at a distance from each other that is difficult to remove [66,67]. In order to cancel the negative charge, we used various coagulants, cations, iron and aluminum that allowed their coagulation and the formation of flocs. Thus, we studied the optimal dose of coagulants like aluminum sulfate, SA, and iron sulfate, IS, for the removal of polyurethane microplastics of various sizes and the time required for their sedimentation.

To improve sedimentation performance, we also studied the influence of flocculants, large molecules, inorganic polymers, polyaluminum chloride, PAC, and non-toxic organic compounds, Aloe Vera gel, AV. We also tried to see if activated carbon influences the removal of microplastics and we used it in combination with the coagulants and flocculants studied.

Figure 3a–d present the turbidity variations in microplastics with the dimensions of D < 100 µm, D1, and 200 µm < D < 500 µm, D2, and in the presence of different coagulants such as aluminum sulfate, polyaluminum chloride, iron sulfate of various concentrations.

From the analysis of Figure 3a,b, it can be seen that the particle size influences the sedimentation process; the larger it is, the higher the turbidity removal efficiency.

The control samples are simple synthetic solutions of PU MPs with sizes D1 and D2, respectively, in water, with an initial turbidity around 175 NTU. The Jar Test shows that after 20 min, the residual turbidity is 47.2 NTU and 17 NTU, respectively, and after 60 min it is 22 and 7 NTU, respectively. The removal efficiency of microplates in the control sample with dimensions D1 and D2 reaches 73% and 90.3%, respectively, after 20 min of sedimentation and 87.5% and 96%, respectively, after 60 min.

Two zones of the sedimentation curve can be distinguished for D2, the first zone, I, between 0 and 20 min when sedimentation occurs rapidly and removes over 90% of the turbidity and the second zone between 20 and 60 min with a slow decrease.

For D1, the first zone is between 0 and 40 min when 85% is removed and the second slow zone with a slow decrease.

Analyzing Figure 3a,b, it can be observed that SA positively influences the removal efficiency of microplastics from PU. The optimal dose required to ensure an efficient settling of microplastic particles with size D1 and D2 is 60 mg L^−1^ sulfates of aluminum, SA.

The removal efficiency of microplastics from PU with D1 reached 94% after 60 min, 9% higher than the control. There was an increase of 5% compared to the control in the first sedimentation zone (first 40 min), and 4% in the second sedimentation zone. The residual turbidity after 60 min is approximately 10 NTU, as shown in Figure 3b.

From Figure 3a, it can be seen that SA causes an increase in the removal efficiency of D2 particles of 95%, approximately 5% higher than the control sample, after 20 min in the first zone, and a final efficiency of 96.6% after 60 min. The residual turbidity is approximately 6 NTU. In the second sedimentation zone, the efficiency increases by 1.6%.

SA was used in doses similar to those used in the study for the removal of microplastics from PS, PE and PVC. Their removal efficiency ranged between 84% and 100% [59,67]. No similar studies were identified for the removal of microplastics from PU.

Aluminum polychloride is not an efficient flocculating coagulant for the removal of microplastics from polyurethane. By adding PAC, the removal efficiency decreases compared to the control sample, so that after the first sedimentation zone the efficiency reaches only 87% compared to 90% for the 20 mg L^−1^ dose, and at the end of the sedimentation, after 60 min, it reaches only 92%, and the residual turbidity is 14 NTU. As the PAC dose increases, the sedimentation efficiency decreases. PAC is not an efficient coagulant for polyurethane microplastics. As we know, PAC is a polymer that has hydroxyl and chloride groups linked to the positive aluminum ion. These groupings probably create repulsive forces with the PU microplastics, which makes sedimentation more difficult. Studies under similar conditions were performed with PAC for PET. Removal efficiency ranged between 33 and 100% [59,67].

From the analysis of Figure 3d, it can be observed that ferrous sulphate does not significantly influence the sedimentation process of PU microplastics. The particles behave similarly to the control sample.

The coagulation process of colloids in the presence of metal ions is determined by the binding of metal ions on the surface of the colloids whose charge is negative, the cancelation of repulsive forces and the formation of smaller or larger flocs depending on the charge of the metal ion. Usually, the higher the electric charge of the metal ion, the larger and heavier flocs can be formed. In order for the flocs to settle, they must have a specific gravity higher than that of water and a large particle size according to Stokes’ law [68]. In the case of using aluminum sulfate, dissociation occurs in aluminum, a metalloid with three positive electrical charges that hydrates in the presence of water and can also form aluminum hydroxide gel. This binds with negatively charged microplastics, canceling their negative charge. The aluminum ion can even bind three microplastics at the same time, forming flocs of different sizes. Studies have shown that the specific gravity of these flocs exceeds the density of water, which leads to an increase in the efficiency of removing PU microplastics from water. The possible mechanism of PU MP removal by coagulation under the influence of SA are shown in Figure 4.

Ferrous sulfate, IS, has a similar behavior, with the difference being that it can bind a maximum of two particles. The key element is the specific gravity, which in this case may not have been higher than the initial mass of the MPs, so the efficiency of the process is not higher than that of the control sample.

The use of PAC does not prove to be beneficial for the coagulation of MPs of PU. Its use has led to an increase in the repulsive forces between the MP colloids, which has led to a slowdown in settling and a decrease in the removal efficiency compared to the control sample.

#### 3.2.2. Identifying the Optimal Dose of Aloe Vera

This stage is meant to identify the influence of some flocculants, in order to reduce the turbidity of contaminated water, by enhancing the aggregation of suspended particles and facilitating solid–liquid separation processes, in order to optimize the removal of polyurethane microplastics.

In addition to the coagulant aluminum sulfate at a concentration of 60 mg L^−1^, Aloe Vera gel, a natural flocculant, was added to the solution in various quantities between 1 g L^−1^ and 3 g L^−1^, to optimize the microplastic removal process, in order to determine the optimal dose to ensure a significant reduction in turbidity.

From the analysis of Figure 5a, it can be noted that the optimal dose necessary to remove microplastic particles with size D2 is 60 mg L^−1^ aluminum sulfate combined with Aloe Vera gel with a concentration equal to 3 g L^−1^, ensuring a residual turbidity of 7 NTU at a retention time of 60 min with an efficiency of 95.2%. Therefore, the combination of SA and AV gel (natural flocculant) has a good effect in reducing turbidity for D2 sizes.

The optimal dose necessary to remove microplastic particles with size D1, is 60 mg L^−1^ aluminum sulfate combined with Aloe Vera gel having a concentration equal to 3 g L^−1^, as shown in Figure 5b. It can be seen that the turbidity efficiency without coagulant for D1 at 60 min is 85.6% (25.2 NTU residual turbidity), and, in the presence of SA with a concentration of 60 mg L^−1^, it reaches 93.1% (10 NTU residual turbidity). In the presence of SA 60 mg L^−1^ and 3 g L^−1^ AV, the residual turbidity was 19.3 NTU, 89.3%. Aloe Vera does not improve the particle removal capacity of D1. The best performance was obtained for SA 60 mg L^−1^.

AV represents a gel with a complex composition that includes vitamins, enzymes, polysaccharides, pectin, lignin, cellulose, and hemicelluloses and others [69]. The active groups in these compounds are those of the hydroxide and carboxyl type that determine a negative charge of the AV molecule. In contact with MPs, this contributes to the increase in the repulsive forces between the particles, partially preventing their settling. At the same time, due to the size of the molecule and the interaction with aluminum ions, on the way to settling, AV also entrains microplastics, ensuring greater efficiency than the free settling of microplastics with size D2.

#### 3.2.3. Identifying the Efficiency of Activated Carbon

The efficiency of granular activated carbon on the removal of microplastics from synthetic solutions was tested. Solutions were prepared in which activated carbon C = 0.5 g L^−1^, aluminum sulfate with a of concentration 60 mg L^−1^, as well as Aloe Vera gel of various concentrations were added.

**Figure 6:**
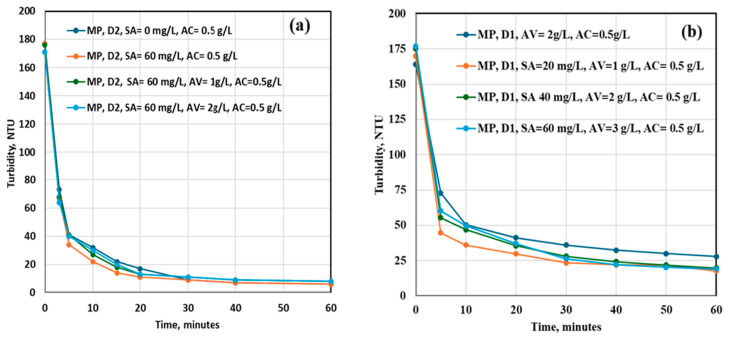
Turbidity variation in microplastics of various sizes in the presence of aluminum sulfate, Aloe Vera and activated carbon: (**a**) 200 µm < D < 500 µm, D2, (**b**) D < 100 µm, D1.

According to Figure 6a, the use of only activated carbon in a concentration of 0.5 g L^−1^ for microplastics with size D2 results in an efficiency of 96.6%, comparable to that obtained in the case of using SA with a concentration of 60 mg L^−1^. The introduction into the system, along with SA 60 mg L^−1^, AC 0.5 g L^−1^ and an AV 1 g L^−1^ for D2, results in a decrease in efficiency, as seen in Figure 6a. This may be caused by a possible competition between microplastics and Aloe Vera for activated carbon adsorption centers. Due to the adsorption of AV, there is an inactivation of active centers. As the amount of AV increases, the microplastic removal efficiency decreases from 95.6% in the system with SA at optimal dose of 60 mg L^−1^ and AV in dose of 1 g L^−1^, to 95.3% for the SA 60 mg L^−1^, AV 2 g L^−1^ and AC 0.5 g L^−1^ system for D2. The introduction of SA, AC or AV into the system causes a rather small decrease in the removal efficiency of microplastics with size D2, ranging between 0.6 and 1.2%. This suggests that they do not represent a problem in terms of sedimentation, and the residual turbidity between 6 and 10 NTU does not represent a problem, because it can be removed by filtration.

For size D1, from the analysis of Figure 6b, it can be seen that the removal efficiency is lower than for those with size D2; the best efficiency is obtained in the presence of SA with a concentration of 60 mg L^−1^, at 94.2%. The introduction of AV with a concentration of 3 g L^−1^ and SA 60 mg L^−1^ decreases the efficiency to 89%. By introducing only AV and AC into the system, the removal efficiency is even lower than in the case of the system without coagulants, reaching 83%. This is explained by the fact that, instead of particle unification, additional repulsion fields are created, and AV and microplastics compete for the activated carbon. Structures are formed that repel each other and are more difficult to remove from the system. The introduction of activated carbon in combination with aluminum SA and Aloe Vera is not an inspired solution. The use of aluminum sulfate is a viable, advantageous solution, because this coagulant is usually used in treatment plants and it has been proven that it is also effective for the removal of microplastics. For polyurethane microplastics of sizes smaller than 100 µm, the residual turbidity can be between 20 and 40 NTU, which can cause water quality problems. These can be found in drinking water, in the residual turbidity that comes out of the filter, at values higher than 1 NTU, which imposes the use of additional or more efficient removal methods.

Activated carbon is biological carbon that is subjected to chemical or physical processes using heat and other chemical agents. Recently, a significant number of published articles have focused on the use of activated carbon as an adsorbent to remove microplastics from wastewater. Activated carbon captures microplastics due to its specific surface and porous structure, which provides numerous active sites for their attachment. The adsorption process is promoted by hydrophobic interactions and van der Waals forces between the microplastics and the activated carbon surface [58,70].

#### 3.2.4. Analysis of Variance and Residuals

The microplastic removal efficiency, Y, was optimized using Response Surface Methodology (RSM), specifically through a Box–Behnken experimental design. A second-order polynomial model was developed, showing a high level of agreement between the adjusted and predicted R^2^ values. The model’s reliability was statistically validated through the analysis of variance (ANOVA), as detailed in Table 2 [71,72]. Mathematical modeling involved estimating the regression coefficients based on data from 17 experimental runs. These coefficients were calculated using Design Expert version 12, which generated the coded form of the quadratic model presented in the following equation.

Y = 93.97 – 1.95 A + 7.00 B + 1.24 C + 1.57 AB – 0.2700 AC – 1.11 BC + 1.36 A^2^ – 3.90 B^2^ + 0.6625 C^2^ (3)

The coefficients of the regression equation have significant implications concerning the comparative influence of the investigated factors on microplastic removal efficiency. The constant term (93.97) represents the baseline efficiency at the central levels of all variables. Among the linear effects, factor B shows the strongest positive contribution (+7.00), confirming its dominant role in enhancing removal efficiency. Factor A exerts a slight negative effect (−1.95), while factor C contributes positively but modestly (+1.24). The interaction terms highlight combined effects: the AB interaction improves efficiency (+1.57), whereas AC (−0.27) and especially BC (−1.11) interactions reduce it. The quadratic terms reveal the non-linear behavior of the system. Factor B^2^ (−3.90) indicates the existence of an optimal range beyond which efficiency declines, while A^2^ (+1.36) and C^2^ (+0.66) show weaker positive curvature effects. Overall, these coefficients emphasize the need to balance factor levels carefully to maximize removal efficiency and demonstrate the practical relevance of the developed model.

To assess the suitability and predictive power of the quadratic model for microplastic removal, an analysis of variance (ANOVA) was conducted. The results, presented in Table 3, demonstrate that the model is statistically robust, with a highly significant *p*-value (<0.0001) and an F-statistic of 76.52, confirming its capacity to account for variations in removal efficiency. According to the data in Table 3, several factors were found to significantly influence the process (*p* < 0.05), including the flow rate (A), microplastic size (B), aluminum sulphate concentration (C), the interaction between flow rate and microplastic size (AB), the interaction between microplastic size and aluminum sulphate concentration (BC), as well as the quadratic terms of flow rate (A^2^) and microplastic size (B^2^). In contrast, the interaction between flow rate and aluminum sulphate concentration (AC), along with the quadratic term of aluminum sulphate concentration (C^2^), did not exhibit a statistically significant effect (*p*-values > 0.05).

The very close fit between the predicted outcomes and the experimental data, along with a high determination coefficient (R^2^), confirms the reliability of the model’s predictive capacity. The ANOVA results further reinforce the appropriateness of the quadratic model in characterizing the decantation process, highlighting the significant influence of flow rate, microplastic particle size, and aluminum sulphate concentration. An Adequate Precision value of 27.8049—well above the recommended minimum of 4—demonstrates a strong signal-to-noise ratio, validating the model’s effectiveness in exploring the design space. The coefficient of variation (C.V. = 0.9243%) is relatively low compared to the standard threshold value of 10%. We can therefore conclude that the experimental work exhibits a high degree of precision and repeatability. A very low C.V. value indicates the stability and reliability of the measurements, which helps minimize random errors. Furthermore, the coefficient of determination (R^2^ = 0.9899), as well as the coefficient of determination adjusted for the number of predictors, known as the adjusted R^2^ (0.9770), indicate a high level of explanation of the variability in the proposed model. These statistical indicators confirm the model’s capacity to reflect the observed data with strong predictive reliability.

**Figure 7:**
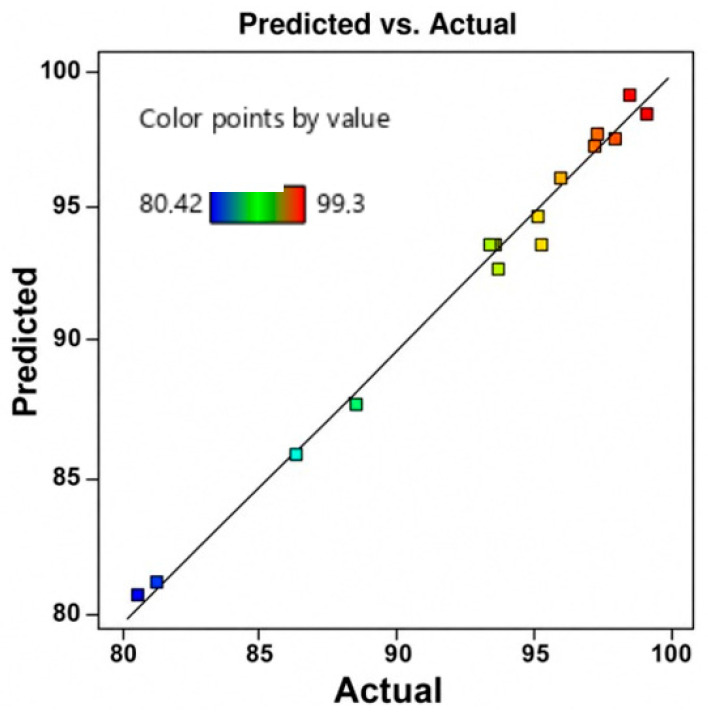
Comparison of the experimental and BBD-RSM-predicted microplastic removal efficiency (%) response.

As illustrated in Figure 7, the residuals exhibit a normal distribution pattern, aligning closely along the reference line. This distribution suggests that the model errors are randomly and symmetrically dispersed, thereby supporting the assumption of normality and reinforcing the statistical validity of the regression analysis.

#### 3.2.5. Graphical Representation of Response Surfaces

**Figure 8:**
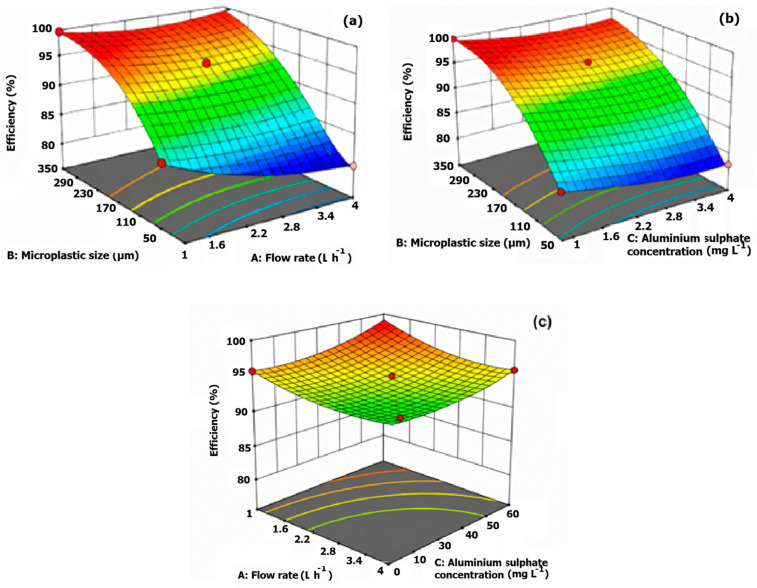
3D response surface plots for microplastic removal efficiency as a function of: (**a**) flow rate and microplastic size, (**b**) microplastic size and aluminum sulphate concentration (**c**) aluminum sulphate concentration and flow rate.

Three-dimensional response surfaces and two-dimensional contour diagrams offer meaningful visual interpretations of how process parameters interact to influence removal efficiency. These graphical representations depict the variation in microplastic removal efficiency (R %) when two variables are simultaneously adjusted, while the third remains fixed at its central value. The contour plots (Figure 8), generated using Design Expert version 12, were instrumental in examining the combined effects of the three selected factors on the response variable Y (%), providing a clearer understanding of their synergistic behavior. The established model equation enables the prediction of response values corresponding to specific levels of the input factors.

Three-dimensional response plots in Figure 8a–c give a close depiction of how the efficiency of microplastic removal is a function of the interaction among important operating parameters. In Figure 8a the combined influence of flow rate (L h^−1^) and microplastic size (µm) indicates that high levels of both variables result in enhanced removal performance. Figure 8b illustrates the interactive effect of microplastic size (µm) and aluminum sulphate concentration (mg L^−1^) with higher aggregation, and removal efficiency noted for higher particle sizes and higher dosages of coagulant. Figure 8c displays the combined effect of aluminum sulphate concentration and flow rate on overall process efficiency. The graph illustrates that efficiency improves under conditions of high coagulant dosage, and relatively low to moderate flow rates. The red to green gradation shift over the surface signifies improving performance that intensifies as increasing amounts of chemicals are added, and maximum efficiency observed where coagulant concentration is high. The findings of these results show that gentle operating conditions, coupled with adequate chemical input, facilitate improved aggregation of microplastics, and subsequently lead to improved removal performance.

#### 3.2.6. Optimization Using Desirability Function

The desirability function is a well-established technique within Response Surface Methodology (RSM) that enables the simultaneous optimization of multiple response variables. In the context of coagulation–flocculation processes, this method converts each response into a dimensionless value ranging from 0 (indicating complete undesirability) to 1 (indicating full desirability). Each response is assigned a desirability score based on how effectively it aligns with the predefined optimization goal typically aimed at enhancing microplastic removal efficiency.

Table 4 shows the optimization criterion, constraints, and optimum solution for microplastic removal. Based on the desirability function approach, the optimal conditions were determined as flow rate of 1.108 L h^−1^, microplastics size of 221.272 µm, and aluminum sulphate concentration of 51.932 mg L^−1^. These conditions correspond to a desirability value of 1, which implies that the optimization objectives were fully achieved.

According to the numerical results obtained, the Smart Decantation-Filtration System, SDFS, is effective in removing microplastics with sizes between 50 µm and 350 µm, from a synthetic solution with a concentration around 175 NTU, and the flow rate in range 1–4 L h^−1^. The flow rate of the raw water supply is a very important parameter influencing the efficiency of the installation. As expected, an increase in plant feed flow rate causes a decrease in turbidity removal efficiency. The turbidity at the outlet of the decanter and at the outlet of the installation was compared with the values imposed by the current legislation Directive 2020/2184/EC and Ordonance 7 of 2023 which impose a maximum permissible limit, MPL, of turbidity 1 NTU.

When using synthetic solutions containing only microplastics with aluminum sulfate in reactants of a 60 mg L^−1^ dose, it was found that the total efficiency of the system ranged between 80.42% and 99.3%. The residual turbidity for the water sample with microplastic particles at the outlet of the decanter has values in the range of 5–20 NTU, after a retention time of 60 min, and after the water is passed through the filter, the residual turbidity has values between 1 and 3 NTU.

The turbidity at the exit of the SDFS decanter is comparable to the turbidity values at the exiting decanters used at industrial level. Turbidity at the exit of the filter of SDFS has values higher than the MPL, which requires either a resizing of the filter which turns out to be undersized, an increase in the retention time in the decanter, or the use of more efficient coagulants and flocculants.

## 4. Conclusions

From the study, it can be seen that the removal efficiency of PU MPs is influenced by the particle size, type of coagulant and flocculant used and the sedimentation time.

The Jar Test was performed at a temperature of 20 °C and an initial pH of 7.2, at an initial concentration of MPs around 175 NTU. The maximum removal efficiency of MPs with D1 size reaches 94.2% and a residual turbidity around 10 NTU for a dose of 60 mg L^−1^ SA; the efficiency is 7% higher than that of the control solution. The efficiency variation curve over time shows that in the first 40 min in zone I, the turbidity decreases rapidly to values over 95% of the removed value. In zone II, from 40 to 60 min the turbidity decreases slowly in percentages of 5% of the total efficiency.

Microplastics with size D2 have a different behavior; the maximum removal efficiency can reach 96.6% in the presence of SA with a dose of 60 mg L^−1^. In the first 20 min in zone I, efficiency increases rapidly to 95% of the initial concentration, and in the remaining 40 min only 1.6% of the initial turbidity is removed; the residual turbidity was 7 NTU. Using SA causes an additional efficiency of 6% compared to the control sample, and 5% of it in Zone I. In this situation, the retention time can be reduced to less than 30 min for larger sizes. The AC used in doses of 0.5 g L^−1^ can achieve a performance similar to SA, for D2. AV reduces the removal efficiency of MPs for both sizes, while the combination of AV and AC is not recommended. The use of iron sulfate has no noticeable influence on the removal capacity of PU MPs, and the use of PAC has a negative influence.

SDFS was designed with many compartments sized so that the retention time in the decanter is at least equal to the sedimentation time required for PU MP particles to be in the presence of various coagulants and flocculants.

For testing the Smart Decantation-Filtration System, three-dimensional microplastics of around 50 µm, around 200 µm and around 350 µm were used. The working conditions were like the Jar Test; synthetic water flow rates were between 1 and 4 L h^−1^, SA dose between 20 and 60 mg L^−1^. The use of aluminum sulphate has a good efficiency on microplastic removal, which can provide a residual turbidity, after a retention time of 50–60 min at 5–20 NTU at the exit of the decanter. This performance can be compared with that of classic decanters used for particle removal in the water treatment process. The decanter is efficient both for synthetic solutions containing only microplastics, and for synthetic solutions with various reagents.

The combined action of the decanter and the filter must ensure an efficiency of removing MPs of less than 1 NTU in the case of drinking water.

The residual turbidity achieved in the SDFS system was between 1 and 3 NTU, higher than the maximum permissible limit, LMA, for water intended for human consumption. Sedimentation in the specially constructed decanter can raise the optimal value of the removal efficiency of polyurethane microplastics to 98.98%, and filtration can ensure an efficiency that reaches over 99.5%.

In conclusion, it is necessary either to identify more efficient flocculating coagulants to further increase the efficiency of the decanter, or to resize the filter in order to reach the values required by the legislation in force.

The experiments must continue so that the SDFS performance is indisputable.

## Figures and Tables

**Table 1:** Technical specifications of Norit GAC 830 W activated carbon [57].

No.	Specification	Active Carbon Tip Norit GAC 830 W
1	Particle size > 2.36 mm	Maximum 15% in mass unit
2	Particle size < 0.6 mm	Maximum 5% in mass unit
3	Moisture	Maximum 5%
4	Iodine number	957 mg g^−1^
5	Methylene blue adsorption	20 g/100 g
6	Ash content	12%
7	Total surface area, BET analyses	1100 m^2^ g^−1^
8	Apparent density	500 kg m^−3^

**Table 2:** Variables and levels considered for microplastic removal.

Variable	Name	Coded Level		
		−1	0	1
		Experimental Values		
A	Flow rate (L h^−1^)	1	2.5	4
B	Microplastic size (µm)	50	200	350
C	Aluminium sulphate concentration (mg L^−1^)	0	30	60

**Table 3:** ANOVA for postulated model.

Source	Sum of Squares	df	Mean Square	F-Value	*p*-Value	
Model	520.80	9	57.87	76.52	<0.0001	Significant
A-Flow rate	30.42	1	30.42	40.23	0.0004	Significant
B-Microplastic size	392.28	1	392.28	518.72	<0.0001	Significant
C-Aluminium sulfate concentration	12.35	1	12.35	16.33	0.0049	Significant
AB	9.86	1	9.86	13.04	0.0086	Significant
AC	0.2916	1	0.2916	0.3856	0.5543	
BC	4.88	1	4.88	6.46	0.0386	Significant
A^2^	7.76	1	7.76	10.26	0.0150	Significant
B^2^	63.96	1	63.96	84.58	<0.0001	Significant
C^2^	1.85	1	1.85	2.44	0.1620	
Residual	5.29	7	0.7562			
Lack of Fit	2.60	3	0.8680			
Pure Error	2.69	4	0.6724			
Cor Total	526.10	16				
Std. Dev.	0.8696			R^2^		0.9899
Mean	93.09			Adj. R^2^		0.9770
CV (%)	0.9342			Pred. R^2^		0.9128
				Adeq precision		27.8049

**Table 4:** Criteria constraints and optimal solutions.

Name	Goal	Lower Limit	Upper Limit	Solution	Desirability
A-Flow rate	is in range	1	4	1.108	1
B-Microplastic size	is in range	50	350	221.272	1
C-Aluminium sulphate concentration	is in range	0	60	51.932	1
Efficiency (%)	maximize	80.42	99.3	98.985	1

## Data Availability

Data can be provided upon request.
